# Tissue spaces are reservoirs of antigenic diversity for *Trypanosoma brucei*

**DOI:** 10.1038/s41586-024-08151-z

**Published:** 2024-10-30

**Authors:** Alexander K. Beaver, Zhibek Keneskhanova, Raúl O. Cosentino, Brian L. Weiss, Erick O. Awuoche, Gretchen M. Smallenberger, Gracyn Y. Buenconsejo, Nathan P. Crilly, Jaclyn E. Smith, Jill M. C. Hakim, Bailin Zhang, Bryce Bobb, Filipa Rijo-Ferreira, Luisa M. Figueiredo, Serap Aksoy, T. Nicolai Siegel, Monica R. Mugnier

**Affiliations:** 1 Department of Pathology, Johns Hopkins School of Medicine, Baltimore, MD USA; 2 Department of Molecular Microbiology and Immunology, Johns Hopkins Bloomberg School of Public Health, Baltimore, MD USA; 3 Division of Experimental Parasitology, Faculty of Veterinary Medicine, Ludwig-Maximilians-Universität München, Munich, Germany; 4 Biomedical Center Munich, Division of Physiological Chemistry, Faculty of Medicine, Ludwig-Maximilians-Universität München, Munich, Germany; 5 Department of Epidemiology of Microbial Diseases, Yale School of Public Health, New Haven, CT USA; 6 Division of Infectious Diseases and Vaccinology, Berkeley Public Health Molecular and Cell Biology Department, Berkeley, CA USA; 7 Gulbenkian Institute for Molecular Medicine, Lisboa, Portugal

**Keywords:** Next-generation sequencing, Gene expression, Parasitic infection, Parasite immune evasion, Immune evasion

## Abstract

The protozoan parasite *Trypanosoma brucei* evades clearance by the host immune system through antigenic variation of its dense variant surface glycoprotein (VSG) coat, periodically ‘switching’ expression of the VSG using a large genomic repertoire of VSG-encoding genes^1–6^. Recent studies of antigenic variation in vivo have focused near exclusively on parasites in the bloodstream^6–8^, but research has shown that many, if not most, parasites reside in the interstitial spaces of tissues^9–13^. We sought to explore the dynamics of antigenic variation in extravascular parasite populations using VSG-seq^7^, a high-throughput sequencing approach for profiling VSGs expressed in populations of *T. brucei*. Here we show that tissues, not the blood, are the primary reservoir of antigenic diversity during both needle- and tsetse bite-initiated *T. brucei* infections, with more than 75% of VSGs found exclusively within extravascular spaces. We found that this increased diversity is correlated with slower parasite clearance in tissue spaces. Together, these data support a model in which the slower immune response in extravascular spaces provides more time to generate the antigenic diversity needed to maintain a chronic infection. Our findings reveal the important role that extravascular spaces can have in pathogen diversification.

## Abstract (web summary)

The dynamics of antigenic variation in extravascular parasite populations using VSG-seq, a high-throughput sequencing approach for profiling variant surface glycoproteins expressed in populations of *Trypanosoma* *brucei*, are explored, showing the role of extravascular spaces in pathogen diversification.

## Main

Every pathogen must contend with the adaptive immune response of its host. *Trypanosoma brucei*, a protozoan parasite and causative agent of human and animal African Trypanosomiasis, has evolved a sophisticated strategy to evade this highly flexible and specific host response^14^. Transmitted by the bite of the tsetse fly, *T. brucei* lives extracellularly in the blood, lymph and interstitial tissue spaces of its mammalian host^15^. To escape clearance by a continuous onslaught of host antibodies, the parasite periodically ‘switches’ expression of its immunogenic variant surface glycoprotein (VSG) coat to new, antigenically distinct variants^1^. With a genomic repertoire of thousands of different VSG-encoding genes^2–5^ and the ability to generate new VSGs through recombination^6,8,16^, the parasite has an enormous capacity for altering its antigenic profile.

Studies examining *T. brucei* antigenic variation in vivo have focused nearly exclusively on parasites in the blood, revealing complex VSG expression dynamics^6–8^. However, it has recently become clear that many, if not most, *T. brucei* parasites inhabit extravascular spaces during both experimental and natural infections^9–13^. Although research has shown that tissue-resident parasites adapt to these environments^11^, cause tissue-specific symptoms^15^ and are associated with increased disease severity^13^, it remains unclear why parasites invade tissue spaces and what role these populations might have in infection.

Several older studies suggested a role for tissue-resident parasites in antigenic variation. These studies found that brain- and lymph-resident parasite populations were antigenically distinct from those in the blood^17–20^, and that antigenic types detectable in the lymphatic fluid could be detected in the blood at later time points during infection^19^. This led these researchers to propose that extravascular spaces might be a site for antigenic variation, with antigenic variants generated in extravascular spaces contributing to systemic immune evasion. However, a later study focusing on very early time points postinfection found no antigenic difference between *T. brucei* populations within the blood and several extravascular spaces, and the community consequently ruled out a role for extravascular parasites in antigenic variation^21,22^. These early investigations were limited by the methodology available at the time, which relied on the use of VSG-specific antisera to analyse parasite antigenic diversity. Modern high-throughput sequencing methods allow VSG expression to be measured accurately and in high resolution^7^. Given the mounting evidence that *T. brucei* parasites persist in and adapt to extravascular spaces, there is a clear need to reinvestigate the dynamics of VSG expression within tissue spaces.

Here, we use VSG-seq^7^, a targeted RNA sequencing (RNA-seq) approach for profiling the VSGs expressed in *T. brucei* populations, to characterize the VSGs expressed by extravascular *T. brucei* parasites. Our results show that extravascular spaces are major reservoirs of antigenic diversity during *T. brucei* infection and that this parasite niche is central to the parasite’s ability to continuously outmanoeuvre the immune system.

## Most VSGs are in extravascular spaces

**Fig. 1:**
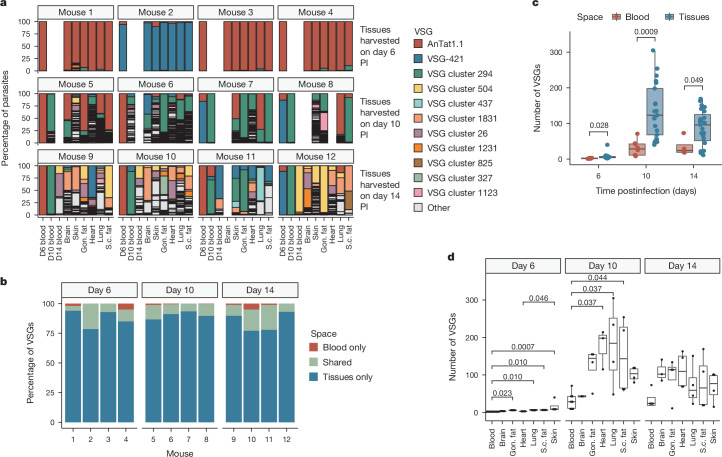
Extravascular parasites harbour most of the antigenic diversity in an infection. **a**, The percentage of parasites expressing each VSG within a space. The 11 VSGs with the highest overall expression are coloured, and all other VSGs are in grey as ‘other’. **b**, Stacked bar graphs from each infected mouse representing the percentage of VSGs that were found exclusively within the blood (red), exclusively within tissue spaces (blue) or shared by both the blood and at least one tissue (green). **c**, Quantification of the number of VSGs found within the blood (red) or tissue spaces (blue) at each time point (Shapiro–Wilk normality test followed by a two-tailed Student’s *t*-test Benjamini–Hochberg corrected). **d**, The number of VSGs in each tissue space (Shapiro–Wilk normality test followed by a two-tailed Dunnett’s test). In **a**–**d**, *n* = 12 total mice with four biologically independent animals per time point over two independent experiments. In boxplots, boxes represent values between the first (25%) and third (75%) quartiles with a line at the median, and extending lines represent the maximum and minimum values not including outliers that are further than 1.5 times the interquartile range. Gon. fat, gonadal fat; PI, postinfection; s.c. fat, subcutaneous fat. Source Data

**Extended Data Fig. 1:**
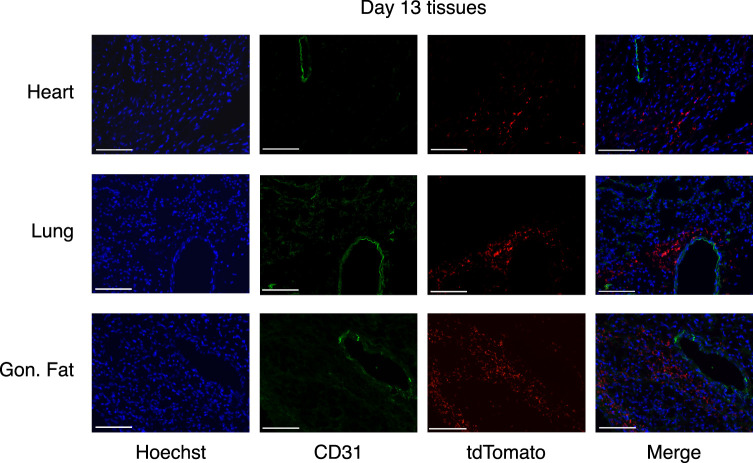
*T. brucei* parasites are found in interstitial tissue spaces. Representative immunofluorescence images of tdTomato expressing parasites (red) from cross sections of perfused tissues stained with hoechst (blue) and anti-CD31 antibody (green). TdTomato-positive parasites localized separately from CD31 lined spaces, showing that parasites are extravascular. n = 3 biologically independent mice examined over three independent experiments. The scale bars represent 100 microns.

**Extended Data Fig. 2:**
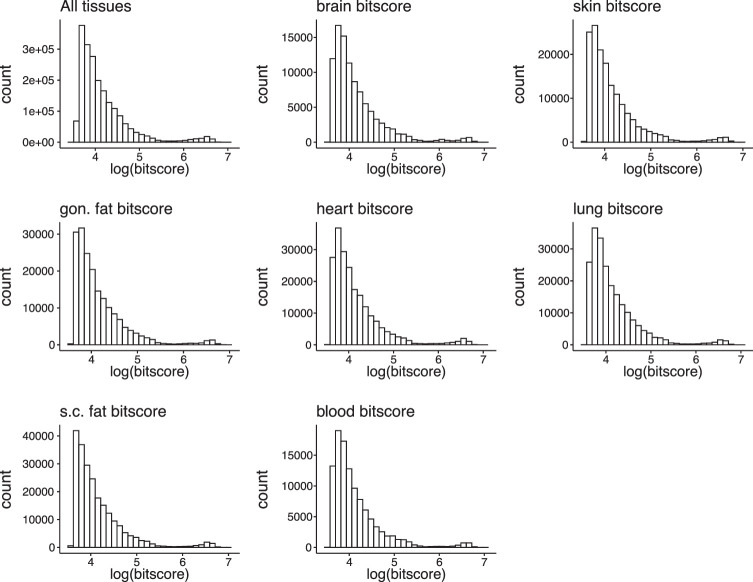
Comparison of VSG sequences expressed by parasites in the blood and tissues. The similarity of VSGs detected in each tissue measured by bitscore, a sequence similarity metric normalized to the database size, allows for comparing tissue compartments with different numbers of total VSGs expressed in each tissue. No statistical significance was found.

**Extended Data Fig. 3:**
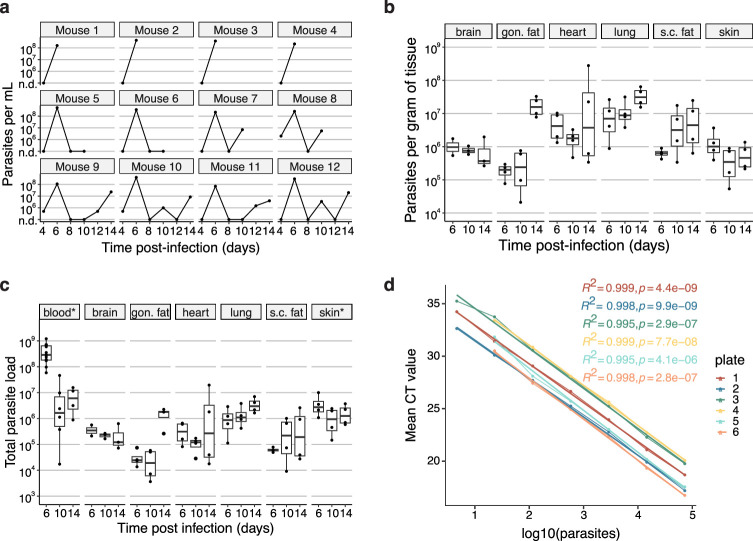
Parasite load in blood and tissues. (**a**) Parasitemia of 12 mice infected with AnTat1.1E *T. brucei* counted from tail blood by hemocytometer (“n.d.” = not detectable, limit of detection of 2.22 × 10^5^ parasites/mL). (**b**) Estimated parasite load per gram of tissue using QPCR. tbZFP3 was used as the control and RNA from known quantities of parasites was used to make standard curves. (**c**) The approximate total number of parasites represented in each organ. This was calculated using the estimated number of parasites from QPCR and the recorded organ mass. For the blood and skin, it was assumed that each mouse had 1.5 mL of blood and 2.73 grams of skin^75^ e point over 2 independent experiments. (**d**) The qPCR standard curves used for each plate of samples. These were used to estimate the number of parasites represented in each tissue sample based on RNA from known parasite concentrations (cultured parasites counted using a hemocytometer). A linear regression model was used to examine correlation between standards, no adjustments for multiple comparisons were made. In boxplots, boxes represent values between the first (25%) and third (75%) quartiles with a line at the median, and extending lines represent the maximum and minimum values not including outliers that are further than 1.5 times the interquartile range. Source Data

**Extended Data Fig. 4:**
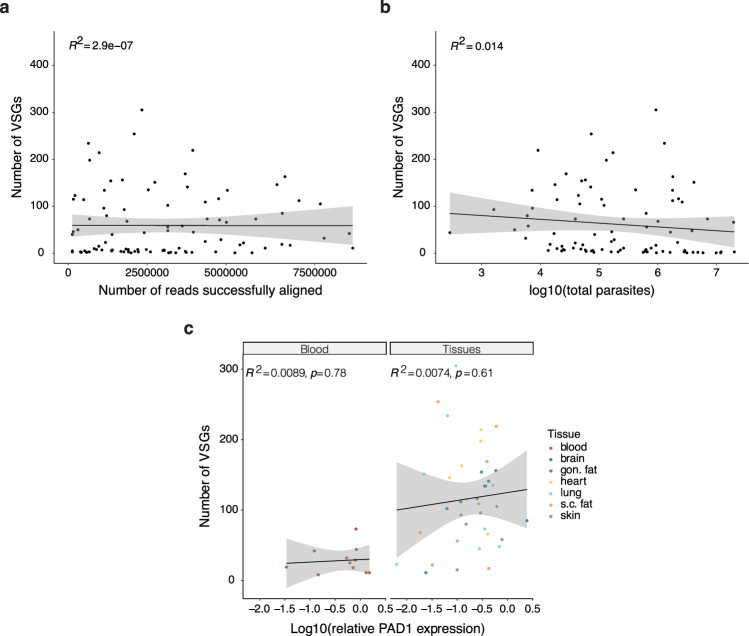
Correlation of VSG counts with read mapping, parasite load, and PAD1 expression. (**a**) A comparison of the number of reads successfully aligned in a sample and the number of VSGs observed. (**b**) A comparison of the total number of parasites and the number of VSGs found in each sample. (**c**) The correlation between PAD1 expression relative to the housekeeping gene tbZFP3 and the number of VSGs expressed at the population level for each sample. Error bands in the above panels represent the 95% confidence interval for predictions from a linear model. Linear regression models were used to calculate R^2^ and P values where noted without adjustments for multiple comparisons. Source Data

To investigate how tissue-resident parasites contribute to antigenic variation in vivo, we intravenously infected 12 mice, each with approximately 5 pleomorphic *T. brucei* EATRO1125 90-13 parasites^23^. We collected blood, then perfused mice with PBS glucose (Extended Data Fig. 1) and harvested the heart, lungs, gonadal fat, subcutaneous fat, brain and skin at 6, 10 and 14 days postinfection. For each sample, we extracted RNA and quantified *T. brucei* VSG expression using VSG-seq^7^. A single ‘initiating’ VSG (either AnTat1.1 or EATRO1125 VSG-421) dominated expression in both the blood and tissues on day 6 (Fig. 1a), in line with previous observations^24,25^. At later time points, VSG expression dynamics became more complex, with more VSGs expressed, a unique composition of VSGs in each tissue, and no single dominating variant (Fig. 1a). Although tissue-specific expression of variant surface proteins is a feature in other organisms that use antigenic variation^26–31^, we found no evidence for tissue-specific VSGs or VSG sequence motifs (Extended Data Fig. 2). Instead, we observed an increase in antigenic diversity in extravascular spaces. The number of detectable VSGs in tissue spaces was, on average, two to four times higher than the blood (Fig. 1c). This did not correlate with parasite load (Extended Data Figs. 3 and 4a,b) and was not driven by any specific tissue (Fig. 1d). In addition, parasite differentiation to the non-dividing tsetse infective form via quorum sensing, which is marked by expression of the *PAD1* gene^32^, did not correlate with VSG diversity in either the blood or tissues at the population level (Extended Data Fig. 4c). The overall contribution of tissue-resident parasites to antigenic diversity in a single infection was large: at any time, roughly 87% of expressed VSGs in any individual infection were found exclusively within extravascular spaces (Fig. 1b).

**Extended Data Fig. 5:**
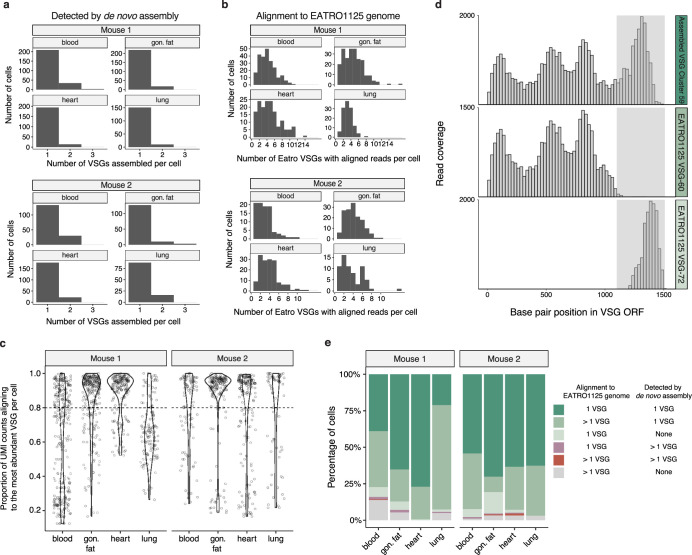
Single-cell sequencing analysis of VSG expression. (**a**) The number of VSG open reading frames (ORFs) detected per cell by de novo assembly with the VSG-Seq pipeline. In this figure, all cells sequenced were evaluated for VSG ORF assembly without any filtering (1377 total cells had a de novo assembled VSG). (**b**) The number of EATRO1125 VSGs detected per single cell. Cells were evaluated for VSG expression only if they had at least 500 genes detected, 1000 gene UMI counts, 30 spike-in UMI counts, and 10 VSG UMI counts (1216 cells fit these criteria out of 2960 total sequenced cells). Only VSGs with >1 UMI count were considered for quantification of VSG expression. These filtering parameters were also applied to genomic mapping analyses in panels **c** and **e**. (**c**) Alignment to the EATRO1125 genome assembly was used to quantify the fraction of VSG UMI counts coming from the most abundant VSG gene within each cell. The dashed line represents the 0.8 fraction of total VSG UMI counts (80%) threshold set to define monogenic expression with one dominant VSG in a cell. (**d**) Representative histograms of coverage for reads from one cell. Read coverage is shown for the de novo assembled VSG cluster 59 and two genomic VSG ORFs, VSG-60 and VSG-72, which represent a common scenario that creates ambiguous VSG expression if reads are only aligned to the EATRO1125 genome. (**e**) The VSG expression classification for each cell using both alignment to the EATRO1125 genome and de novo assembly. In alignment and assembly, if a VSG represented 80% or more of the VSG UMI counts in a cell (for genomic mapping) or 80% of the population (for VSG-seq analysis), that cell was considered to be expressing only the dominant VSG. Source Data

VSG-seq is a bulk measure of VSG expression. To be sure that the increased VSG diversity we observed in tissues was not due to the derepression of silent VSGs within individual cells, we performed single-cell RNA-seq using the SL-Smart-seq3xpress platform to quantify VSG expression in single cells from blood and tissue samples^33^. Because *T. brucei* can form new ‘mosaic’ VSGs through recombination^6,8,16,34^, which are, by definition, absent from reference genomes, we initially used our VSG-seq pipeline to de novo assemble VSGs in each cell. This approach accounts for the possibility that expressed VSGs might be absent from the reference genome, either as a result of mosaic VSG formation or as a result of an incomplete genome assembly, potentially affecting quantification and/or read mapping. Only one VSG assembled in most cells (94.2%; Extended Data Fig. 5a). We also mapped sequencing reads to the EATRO1125 genome, which could reveal more subtle signatures of derepression. By this analysis, there was no obvious difference between the blood and the tissues in the number of expressed VSGs in each cell (Extended Data Fig. 5b) or in the relative expression of the most abundant VSG in each cell (Extended Data Fig. 5c).

To estimate the number of cells maintaining monogenic expression, we defined a cell as maintaining monogenic expression if 80% of unique molecular identifiers (UMIs) mapping to VSGs mapped to a single VSG. Although mapping to the genome suggested that most cells (62.7%) maintained monogenic expression based on this threshold, the proportion of cells estimated to maintain monogenic expression was lower than estimated by de novo assembly. Comparison of the two analyses showed that of those cells expressing more than one VSG by genome alignment, 85% were found to express only one VSG by mapping to the de novo assembled VSGs and using the same 80% threshold for defining monogenic expression (Supplementary Data 1–3). Further investigation revealed that in most of these cases (95.3%) the alignment to many genomic VSGs was an artefact, where the assembled VSG was not well represented within the annotated sequences of the EATRO1125 genome or there were several VSGs with high similarity to the assembled VSG, leading to inaccurate VSG expression quantification (Extended Data Fig. 5d). We estimate that 93.4% of cells were probably expressing only one VSG, with no bias for multigenic VSG expression in tissue spaces (Extended Data Fig. 5e, shades of green, and Extended Data Table 1). In 5.2% of cells, reads mapped to several EATRO1125 VSGs, but no VSG could be assembled (grey). Although it is impossible to distinguish between multi- and monogenic VSG expression in this set of cells, the proportion of cells in this category did not differ between blood and tissues. The few cells that seem to express more than one VSG (1.05% of cells in the blood and 1.03% of cells in the tissues) could indicate sorting doublets or could represent cells mid-switch (Extended Data Fig. 5e, red and purple). Overall, these data indicate that VSG monogenic expression is maintained by most cells in both extravascular spaces and the blood and that the increased VSG diversity we observe in tissue spaces is unlikely to be due to the specific derepression of silent VSGs in tissue populations.

## Common VSGs appear in tissues first

**Fig. 2:**
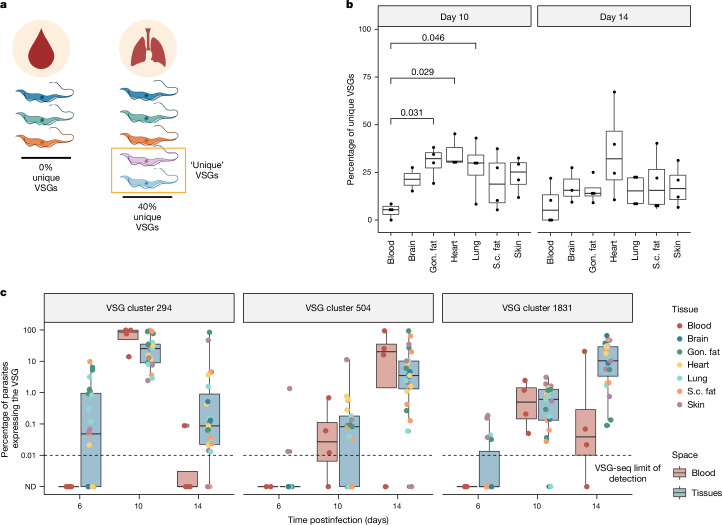
Tissue-resident parasites express a unique repertoire of VSGs during infection. **a**, We define ‘unique’ VSGs as those VSGs solely found within a specific space in a mouse. **b**, The percentage of VSGs that were unique to one space within a mouse (Shapiro–Wilk normality test followed by a two-tailed Dunnett’s test). Day 6 samples were excluded from this analysis because few VSGs are expressed at this point. *n* = 4 biologically independent animals per time point over two independent experiments (total of eight mice). **c**, The expression of three representative VSGs (cluster 294, 504 and 1831) within blood and tissue samples on days 6, 10, and 14. *n* = 12 total mice with four biologically independent animals per time point over two independent experiments. ND indicates that the VSG was not detected. In boxplots, boxes represent values between the first (25%) and third (75%) quartiles with a line at the median, and extending lines represent the maximum and minimum values not including outliers that are further than 1.5 times the interquartile range. Illustration in **a** created using BioRender (https://biorender.com). Source Data

The high antigenic diversity observed in tissues could serve to maintain a chronic infection. If antigenic variation occurs relatively rarely in the bloodstream, then parasites from extravascular spaces might serve as a source of new, antigenically distinct, VSGs. In line with this, tissue spaces contain more ‘unique VSGs’, those VSGs that are expressed exclusively within one space in an infection, than the blood (Fig. 2a,b). To examine the potential for tissue-resident VSGs to contribute to antigenic variation systemically, we identified VSGs only expressed in tissues on day 6 postinfection and analysed whether they later appeared within the blood. Most (74%) of these VSGs were expressed within the blood on day 10 or 14. Analysis of individual VSGs revealed that rare VSGs expressed at low levels exclusively in tissue spaces also have the potential to become ubiquitously expressed within a host (Fig. 2c). In addition to a model in which tissue spaces provide new VSGs to re-seed the blood, it is possible that tissue-resident parasites undergo antigenic variation before blood-resident populations. Therefore, these data could be explained by either a trigger within the tissue environment that induces parasite switching or a differential selective pressure imposed by the tissue environment.

## Parasite clearance is delayed in tissues

**Fig. 3:**
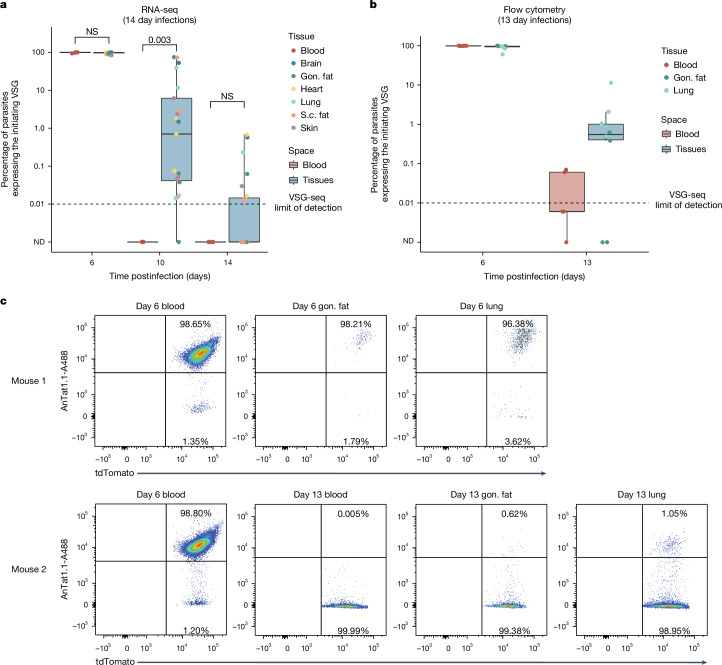
VSG-specific parasite clearance is slower in tissues than in the blood. **a**, The percentage of parasites expressing the initiating VSG (AnTat1.1 or VSG-421) at days 6, 10 and 14 postinfection. Tissue samples were grouped together (blue) and compared to blood samples (red) (two-tailed Wilcoxon test). *n* = 12 total mice with four biologically independent animals per time point over two independent experiments. **b**, Quantification of the number of parasites that were tdTomato positive and stained positive for AnTat1.1 by flow cytometry (*n* = 10 total mice with five biologically independent mice per time point examined over one independent experiment). The horizontal dotted line represents the limit of detection for VSG-seq. **c**, Representative flow cytometry plots from tissues collected from mice infected with chimeric triple-marker parasites that express tdTomato constitutively in their cytoplasm. Parasites were stained with anti-AnTat1.1 antibody. In boxplots, boxes represent values between the first (25%) and third (75%) quartiles with a line at the median, and extending lines represent the maximum and minimum values not including outliers that are further than 1.5 times the interquartile range. NS, not significant. Source Data

Indeed, our data suggest that the environment within tissue spaces is distinct from the blood, with parasite clearance occurring at different rates in each space. Although parasites expressing the initiating VSG were cleared from the blood by day 10 postinfection, they were not cleared from tissues until at least day 14 (Fig. 3a). This suggests that VSG-specific parasite clearance from extravascular spaces is delayed, but not abolished, compared to the blood. VSG-seq is a measure of VSG expression at the transcript level, however. To confirm this observation at the protein level, we performed flow cytometry on *T. brucei* cells from the blood, lungs and gonadal fat, using the tdTomato-expressing ‘triple reporter’ *T. brucei* EATRO1125 AnTat1.1E cell line^35^ (Fig. 3b,c and Extended Data Table 2). The flow cytometry analysis showed a detectable AnTat1.1-expressing tissue parasite population at day 13 postinfection, a time point at which AnTat1.1^+^ parasites were undetectable, or nearly undetectable, in the blood. Similar to the increase in antigenic diversity we observed in every tissue space, this delay in clearance, observed at both the RNA and protein levels, was not tissue-specific. Thus, the immune mechanisms influencing extravascular parasite clearance seem to be general features of extravascular spaces.

## High diversity after infection by tsetse

**Fig. 4:**
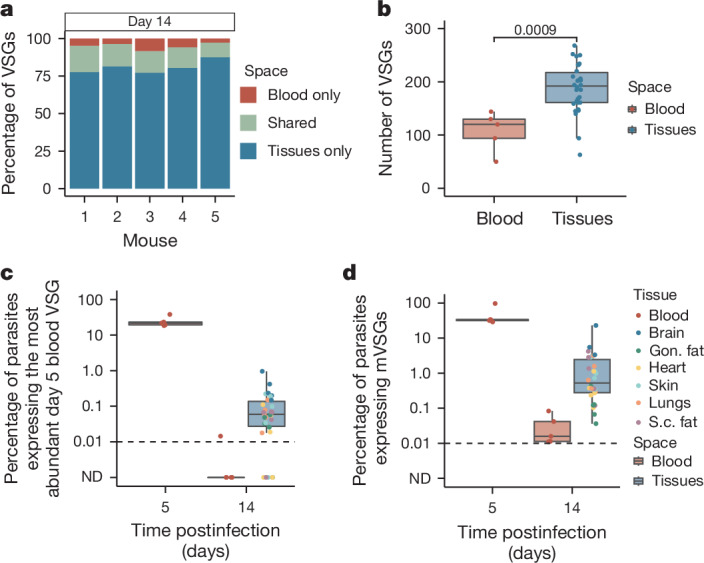
Tsetse bite-initiated infections show increased antigenic diversity and delayed immune clearance in extravascular spaces. Data from five mice infected with RUMP 503 parasites from a tsetse fly bite. **a**, Bar graphs representing the percentage of VSGs in each mouse that were found exclusively within the blood (red), exclusively within tissue spaces (blue) or shared by both the blood and at least one tissue (green). **b**, Quantification of the number of VSGs found within the blood (red) or tissue spaces (blue) on day 14 postinfection (Shapiro–Wilk normality test followed by a two-tailed Student’s *t*-test Benjamini–Hochberg corrected). **c**, The percentage of parasites within each mouse expressing the most abundant VSG from the day 5 blood. **d**, The percentage of parasites expressing one of the mVSGs found to be expressed by RUMP 503 parasites in the salivary gland of a tsetse fly. In boxplots, boxes represent values between the first (25%) and third (75%) quartiles with a line at the median, and extending lines represent the maximum and minimum values not including outliers that are further than 1.5 times the interquartile range. For **a**–**d**, *n* = 5 biologically independent mice examined over one independent experiment. Source Data

A benefit of starting infections with a small intravenous inoculum is that it creates convenient and reproducible infections. In nature, however, a tsetse fly bite introduces thousands of parasites into the skin, each expressing a single metacyclic VSG (mVSG) from a limited repertoire^36^. To ensure that our observations held true in this more complex context, we repeated our infections using a more natural tsetse bite infection model. Infections were initiated in five mice by tsetse bite using flies infected with RUMP 503 *T. brucei* parasites^37^. We used VSG-seq to quantify VSG expression in the blood on day 5 postinfection and the blood and tissues on day 14 postinfection. In line with our previous observations, we found that in tsetse-initiated infections roughly 80% of VSGs were exclusively expressed within extravascular spaces (Fig. 4a) and tissue populations harboured more VSGs than the blood (Fig. 4b). This demonstrates that extravascular spaces are the primary reservoir of antigenic diversity, even when infections are initiated by fly bite.

Parasite populations were more diverse at early time points in tsetse infections than intravenous infections, probably due to the larger and more heterogeneous inoculum delivered by the fly. To analyse VSG-specific parasite clearance in tissues, we quantified expression of the most abundantly expressed VSG in the blood of each mouse on day 5 as well as the known mVSG repertoire from this parasite strain, which should represent the repertoire of VSGs expressed at the start of an infection. In both cases, we found that on day 14, tissue spaces still contained parasites expressing the most abundant VSG and/or mVSGs, whereas these VSGs were expressed by no or very few parasites in the blood (Fig. 4c,d). This suggests that in tsetse bite-initiated infections, as we observed in intravenous infections, tissue parasite populations are cleared at a slower rate than parasites in the blood.

## Delayed clearance increases diversity

**Fig. 5:**
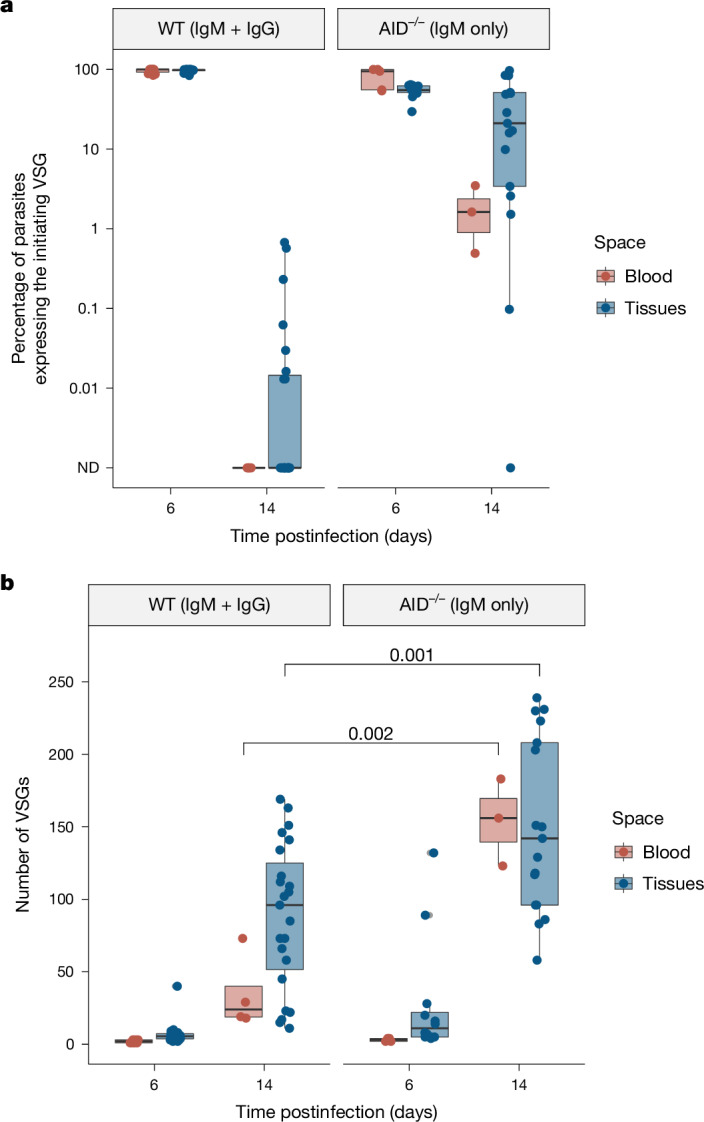
Delayed parasite clearance correlates with an increase in VSG diversity. **a**, The percentage of parasites expressing the initiating VSG (AnTat1.1 or VSG-421) in both wild-type (WT) and AID^−/−^ mice. **b**, The number of VSGs expressed within the blood (red) and tissues (blue) of WT and AID^−/−^ mice (Shapiro–Wilk normality test followed by a two-tailed Student’s *t*-test). For WT mice, *n* = 12 total biologically independent mice with four mice representing harvested tissues for each time point. For AID^−/−^ experiments, *n* = 5 total biologically independent mice with tissues from two mice represented on day 6 and tissues from three mice represented on day 14. Day 6 blood represents samples from all five mice. In boxplots, boxes represent values between the first (25%) and third (75%) quartiles with a line at the median, and extending lines represent the maximum and minimum values not including outliers that are further than 1.5 times the interquartile range. Source Data

**Extended Data Fig. 6:**
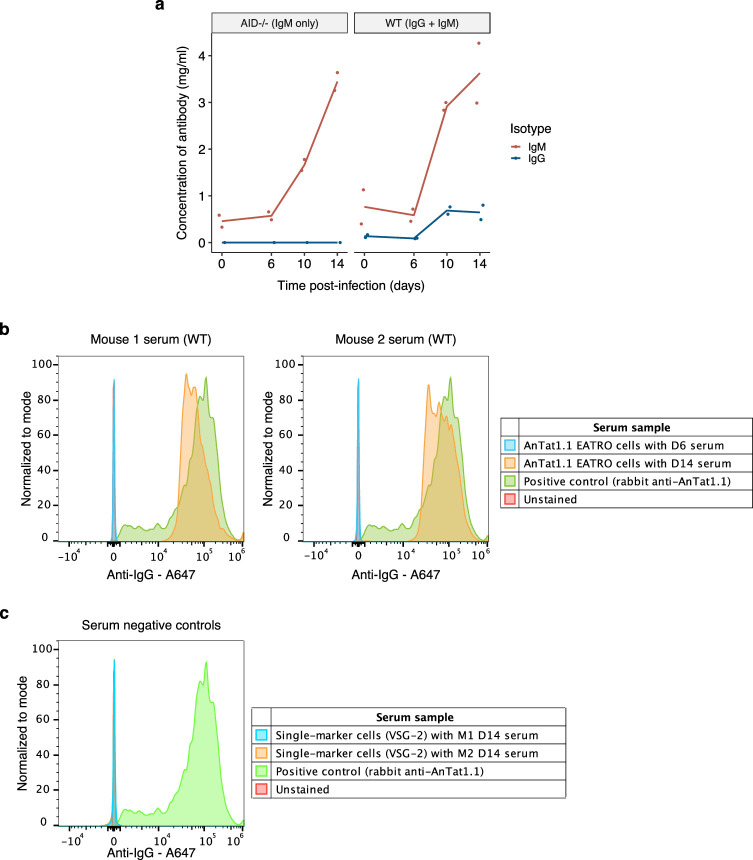
General and VSG-specficic antibody levels during infection. (**a**) Quantification of serum IgM and IgG concentrations in infected AID^−/−^ and wildtype (WT) mice by ELISA (n = 2 biologically independent animals per group over one independent experiment). (**b**) AnTat1.1 EATRO cells (expressing VSG AnTat1.1) stained with serum, followed by an anti-IgG secondary antibody that cross-reacts with IgM and other isotypes, from day 6 and day 14 of two mice infected with AnTat1.1 Triple-marker cells. A polyclonal rabbit anti-AnTat1.1 antibody on cells known to be expressing AnTat1.1 was used as a positive control. (**c**) Single-marker cells (expressing VSG-2) stained with day 14 serum from two mice infected with AnTat1.1 Triple-marker cells. These were used as negative controls to show that serum from infected mice specifically binds AnTat1.1 and no other VSGs. Source Data

The increased antigenic diversity in extravascular spaces could be explained by the distinct clearance dynamics we observe, as prolonged survival in tissues could provide more time for parasites to switch. To test whether there was a link between parasite survival and increased diversity, we sought to interrupt parasite clearance in tissues. Because parasite clearance in the blood coincides with the appearance of anti-VSG IgM, between days 8 and 10, and parasite clearance in the tissues coincides with the anti-VSG IgG response, between days 10 and 14 (refs. ^1,12,38–41^), we proposed that clearance in tissues is dependent on the anti-VSG IgG response. Thus, the loss of IgG might abrogate the clearance of tissue-resident parasites. To test this hypothesis, we infected activation-induced cytidine deaminase (AID) knockout (AID Cre) mice^42^, which only produce IgM antibodies (Extended Data Fig. 6), and analysed blood and tissues from days 6 and 14 postinfection by VSG-seq. As expected, clearance of the initiating VSG was severely delayed in tissues on day 14 in AID^−/−^ mice, suggesting that IgG is important, if not critical, for the clearance of extravascular parasites. This could be explained by the fact that IgM, a bulky pentamer, does not diffuse efficiently into tissue spaces^43,44^, whereas IgG, a monomer, readily diffuses. We also observed a defect in the clearance of blood-resident parasites in AID^−/−^ mice compared to the blood of wild-type mice (Fig. 5a). In both the blood and tissues of AID^−/−^ mice, where clearance was delayed, more VSGs were detected on day 14 postinfection compared to wild-type (Fig. 5b), showing a direct relationship between the timing of parasite clearance and VSG diversity. Regardless of their local environment (intra- or extravascular), longer-lived parasite populations generated more diverse sets of VSGs.

## Discussion

The idea that antigenic variation might occur outside the bloodstream, with extravascular populations contributing to immune evasion within the bloodstream, is not a new one. Here, using modern high-resolution techniques, we provide evidence for this long-standing hypothesis. In both needle- and tsetse bite-initiated infections, we find that extravascular spaces are the primary reservoir of VSGs, accounting for most antigenic diversity in any individual infection. The number of VSGs we detected in the blood matches previous estimates^6–8^, whereas the diversity in tissues is two to four times higher. Our data indicate that this is at least partially due to slower clearance dynamics in extravascular spaces and highlight the role that tissue spaces can have in pathogen diversification.

Although extravascular parasite populations were highly antigenically diverse, we saw no evidence of tissue-specific VSG expression. Because parasites invade tissues efficiently before much VSG switching has occurred, it seems unlikely that any specific VSG is required for tissue invasion. Whether VSGs could influence parasite fitness in specific host spaces is less clear. We measured VSG expression up to day 14 postinfection, at which point tissue-resident populations are just beginning to diverge from one another and the blood. It is therefore possible that, as these populations further evolve, there may be a selection for VSGs better adapted to certain tissue spaces.

It has previously been shown that in the bloodstream alone *T. brucei* expresses more VSGs than seem to be required for immune evasion^7^. Here we find that the expressed VSG diversity within host tissues is even greater. Although on the surface it could seem to be disadvantageous for *T. brucei* to use so many different antigens this quickly, this striking diversity could be important for the parasite. In natural infections, particularly in wild animals in which pre-existing anti-VSG immunity is more likely to exist, a high switch rate may be required to ensure some parasites successfully evade the host’s existing antibody repertoire. Moreover, infections in the wild can last for months to years^14,45^. During these long infections, the large reservoirs of VSGs found in tissues may be essential for the maintenance of a chronic infection.

Indeed, our data support the idea that the large reservoirs of antigenic diversity in extravascular spaces contribute to systemic infection when parasites re-enter the blood after switching: rare VSGs expressed exclusively in tissues at early time points are expressed in the blood and other spaces later. This is also in line with another recent study, which showed that blood-resident parasites are largely non-replicative, indicating that tissue-resident parasites may be required to re-seed the blood^46^. There is another intriguing explanation for this observation, however. Switching in *T. brucei* is known to be semipredictable^7,47^, and it is possible that tissue-resident parasites simply switch earlier, or more frequently, than those in the blood. In this case, the same VSGs would arise independently in every population without any parasite movement between spaces. Whereas the high vascular permeability observed after the initial stages of infection indicates that parasites probably move back and forth between the vasculature and extravascular spaces, the fact that tissue-resident populations contain many unique VSGs suggests that blood re-entry may represent a bottleneck for the parasite. An increased rate of switching in tissues could be explained by a higher proportion of dividing slender form parasites in these spaces, as has been observed in the adipose tissue^13^, but we found no correlation at the population level between *PAD1* expression and increased VSG diversity. Notably, increased diversity is still observed when the overall parasite load in tissues is lower than the blood (Extended Data Fig. 3c). It is therefore exciting to speculate that some aspect of the extravascular environment supplies a molecular or physical stimulus that promotes VSG switching.

Whereas the timing of VSG switching could be the result of an environmental trigger, our data indicate that the higher antigenic diversity in extravascular spaces compared to the blood can also be explained, at least in part, by the dynamics of the immune response to *T. brucei* in each space. In tissue spaces, we observed slower VSG-specific clearance of parasites than in the blood, potentially providing these populations more time to undergo antigenic variation. Furthermore, newly switched parasites are still vulnerable to immune clearance by antibodies against their previous VSG for roughly 29 h, so moderate delays in immune clearance could allow more switched parasites to survive^48^. The direct relationship we observed between the timing of parasite clearance and antigenic diversity in AID^−/−^ mice supports this model, with even small delays in clearance showing great effects on parasite VSG diversity in both the tissues and the blood.

Our results show that the production of IgG has a key role in clearing parasites from tissues, which we propose could be related to the ready diffusion of this molecule within extravascular spaces facilitating parasite clearance. It is important to note, however, that whereas the difference in timing between the anti-VSG IgM and IgG responses inspired the hypothesis that IgG might be important for parasite clearance in tissues, our study does not prove that this difference in timing explains the delayed clearance in tissue spaces. The local immune response to *T. brucei* is complex, with many mechanisms likely to have a role in parasite detection and clearance; our results indicate that IgG is an important player in this response. The increased VSG diversity we observe is certainly multifactorial and could be influenced by parasite factors, such as metabolism, division, motility and antibody internalization, and host factors, such as extravascular environmental stresses, the local immune response and vascular permeability. More research will be required to fully understand the complex nature of the *T. brucei* host–pathogen interaction within the extravascular niche.

Altogether, our results outline a model in which *T. brucei* parasites ‘hide’ in extravascular spaces to generate new antigenic variants capable of exiting tissues and aiding in systemic immune evasion. Coupled with other recent studies^9–13,46,49^, this suggests a framework for the progression and pathogenesis of *T. brucei* infections where, instead of being the primary parasite reservoir, the blood may represent a transient population that is regularly reseeded by extravascular parasites. The vasculature, then, might act as a highway system for movement between the tissue spaces and for eventual transmission back into the tsetse fly.

Interfering with the egress from or establishment within tissue spaces might be a strategy for treating *T. brucei* infections or other infections with pathogens that rely on the distinct features of the extravascular environment. In line with this, one recent study showed that partial inhibition of *T. brucei* tissue invasion using antibodies against P- and E-selectins results in prolonged survival in mice^13^. This also fits with data from another group that found immotile *T. brucei* parasites, probably unable to invade tissues, were no longer infectious^50^. It is possible that without the proper establishment of parasite tissue reservoirs, overall antigenic diversity is lowered, limiting the parasite’s capacity for immune evasion and leading to a decrease in parasite burden. Defining the dynamics and variation of parasites both within and between spaces, as well as the unique host environment within each tissue space, will be central to understanding how *T. brucei* consistently avoids immune clearance and harnessing this mechanism for disease control.

More broadly, these data demonstrate how different environmental and immune pressures within a host can influence pathogen diversification. The production of genetic heterogeneity within an infection is important for many pathogen virulence processes, including establishing and maintaining infection, facilitating immune evasion, generating drug resistance and adapting to different host environments. The extravascular environment has a unique role in promoting pathogen evolution, and *T. brucei* serves as a valuable model for understanding this aspect of the host–pathogen interface.

## Methods

### Intravenous mouse infections and sample collection

Female C57Bl/6J (wild-type, strain no. 000664 Jackson Laboratory) or B6.129P2-*Aicda*^*tm1(cre)Mnz*^/J (AID^−/−^, strain no. 007770 Jackson Laboratory)^42^ between 7 and 10 weeks old were housed at 68–76 °C (target 72 °C) with 30–70% relative humidity (target 42%) under a 14.5 h:9.5 h light:dark photoperiod. Mice were infected by intravenous tail vein injection with roughly five pleiomorphic EATRO1125 AnTat1.1E 90-13 *T. brucei* parasites^23^. Blood parasitaemia was counted by tail bleed every 2 days starting on day 4 postinfection by haemocytometer with a limit of detection of 2.22 × 10^5^ parasites per millilitre. Blood (25 μl) was collected by a submandibular bleed on days 6, 10 and 14 postinfection and placed into TRIzol LS. For wild-type mice, four mice were anaesthetized and perfused at days 6, 10 and 14 postinfection. Infected AID^−/−^ mice were anaesthetized and perfused at days 6 (two mice) and 13 (three mice) postinfection. Sample sizes were chosen based on preliminary data and for experiment feasibility. For all experiments, mice were assigned to each collection time point randomly from the full set of mice; mice were not housed by sample group. Samples were not blinded at any point. Mice were perfused with 50 ml of PBS glucose (0.055 M d-glucose) with heparin. After perfusion, tissues were dissected and placed immediately into 1 ml of RNA Later. The heart, lungs, gonadal fat, subcutaneous fat, brain and skin (ear) were collected.

For flow cytometry, immunofluorescence experiments and single-cell sorting experiments, 7–10-week-old female C57Bl/6J mice were infected by intravenous tail vein injection with roughly five AnTat1.1E chimeric triple reporter *T. brucei* parasites that express tdTomato^35^. Blood was collected by a submandibular bleed at designated time points. For flow cytometry, mice were anaesthetized and perfused on days 6 and 13 postinfection as discussed above and the gonadal fat and lungs were harvested. For immunofluorescence experiments, perfused tissues were collected at day 13 postinfection. For single-cell sorting, blood and tissues were only collected on day 14 postinfection. All animal studies were approved by the Johns Hopkins Animal Care and Use Committee (protocol no. MO22H163).

### VSG-seq sample and library preparation

RNA was isolated from blood samples stored in TRIzol LS (ThermoFisher, catalogue no. 10296010) by phenol–chloroform extraction. Tissue samples were weighed and homogenized in TRIzol, and then RNA was isolated by phenol–chloroform extraction. RNA from each sample was DNase treated using Turbo DNase and cleaned up with Mag-Bind TotalPure NGS beads (Omega Bio-Tek, M1378-00). First-strand complementary DNA (cDNA) synthesis was performed using SuperScript III Reverse Transcriptase and a primer that binds to the conserved VSG 14-mer in the 3′ untranslated region (5′-GTGTTAAAATATATC-3′). Products were cleaned up using Mag-Bind TotalPure NGS beads (Omega Bio-Tek, M1378-01). Next, a VSG-specific PCR with Phusion polymerase (ThermoFisher, F530L) was performed using primers for the spliced-leader (5′-ACAGTTTCTGTACTATATTG-3′) and SP6-VSG 14-mer sequences (5′-GATTTAGGTGACACTATAGTGTTAAAATATATC-3′) for 25 cycles. VSG-PCR products were cleaned up using Mag-Bind TotalPure NGS beads and quantified using the QuBit HS DNA kit (Life Technologies). Finally, sequencing libraries were prepared with the Nextera XT DNA Sample Prep Kit (Illumina) using the manufacturer’s guidelines, and libraries were sequenced with 100 bp single-end reads on an Illumina HiSeq 2500.

### Tissue-load and *PAD1* qPCRs

First-strand synthesis was performed with SuperScript III Reverse Transcriptase (ThermoFisher Scientific, 18080051) and random hexamer primers on tissue RNA samples. Quantitative PCR (qPCR) was performed in triplicate using SYBR Green qPCR Master Mix (Invitrogen, 4309155). *TbZFP3* primers were used to estimate parasite load in tissue samples (forward 5′-CAGGGGAAACGCAAAACTAA-3′; reverse 5′-TGTCACCCCAACTGCATTCT-3′). Cycle threshold (Ct) values were averaged between the triplicates and parasite loads per mg of tissue were estimated using a standard curve of values from RNA isolated from known numbers of cultured parasites (standard curves can be found in Extended Data Fig. 3d).

For *PAD1* expression quantification, RNA extraction, first-strand synthesis and qPCR were performed following the same methods as above. *PAD1* expression was quantified by normalizing to *TbZFP3* as a control gene (same primers as above) (*PAD1* primers; forward 5′-CAGCGGCGATTATTGCATTGG-3′; reverse 5′-AGGAAGAAGGTTCCTTTGGTC-3′). Ct values were averaged between the triplicates and samples were compared using the delta-CT between *PAD1* and *TbZFP3*.

### VSG-seq analysis

Analysis of sequencing results was performed following the method we reported previously^7^, with two changes: no mismatches were allowed for bowtie alignments and each sample was analysed (assembly, alignment and quantification) separately. The data were analysed using the VSG-seq pipeline available on GitHub (https://github.com/mugnierlab/VSGSeqPipeline, commit 226a8a1b3cc050391c6e62f9ababc3594177d0dd). The following software and versions were used in the pipeline: Trinity^51,52^ (v.2.8.5), Bowtie^53^ (v.1.2.3), Biopython^54^ (v.1.72), Blast^55,56^ (v.2.9), Bedtools^57^ (v.2.29.2), cd-hit^58,59^ (v.4.8.1), trim-galore^60^ (v.0.6.4) and samtools^61^ (v.1.9). To compare expressed VSG sets between samples, all assembled VSGs were clustered using CD-HIT-EST^58,59^ (v.4.8.1). VSGs with more than 98% identity to one another were conservatively treated as one VSG. VSGs were then identified by their Cluster number for further analysis. Samples that had less than 100,000 successfully aligning reads to VSGs were excluded from further analysis. Four samples, three brain and one heart, were discarded because fewer than 100,000 reads aligned to VSG (Extended Data Fig. 3a). Downstream analysis of expression data and generation of figures was performed in R v.4.3.1.

### Analysis of VSG sequence motifs

To identify whether there were tissue-specific VSG sequence motifs, the similarity of N-terminal sequences from all assembled VSGs were compared. N-terminal sequences were identified using a HMMEr scan^62^ (v.3.1b2) against a database curated by Cross et al.^2,63^. All N termini were compared in an all versus all BLAST^55,56^ (v.2.9) using default parameters. All VSG pairwise comparisons with an e-value higher than 1 × 10^−3^ were considered sufficiently similar to one another for further analysis. VSGs that were found in a given tissue were binned into that tissue group, and the distribution of the BLAST bitscores in a given compartment was compared against the total population of similar VSGs.

### Flow cytometry

Once mice were perfused, tissues were dissected and washed with Hank’s balanced salt solution (ThermoFisher Scientific, 14175095). Tissue samples were minced and placed in DMEM (ThermoFisher Scientific, 11995065) containing either 1 mg ml^−1^ collagenase type 1 (ThermoFisher Scientific, 17100017) for adipose fat or 2 mg ml^−1^ collagenase type 2 (ThermoFisher Scientific, 17101015) for lung samples. Hearts were dissociated using 2 mg ml^−1^ collagenase type 2, 50 U ml^−1^ DNase I and 20 U ml^−1^ hyaluronidase. These were then incubated in a 37 °C water bath for 1 h and briefly vortexed every 10 min. Next, samples were passed through a 70 µM filter and centrifuged at 2,600*g* for 8 min at 4 °C, and the cell pellet was taken for antibody staining.

Blood samples were collected by submandibular bleed and red blood cells were depleted by magnetic-activated cell sorting with anti-Ter-119 MicroBeads (Miltenyi Biotech, 130-049-901) following the manufacturer’s protocol. Cells were pelleted and washed with HMI-9 media.

**Extended Data Fig. 7:**
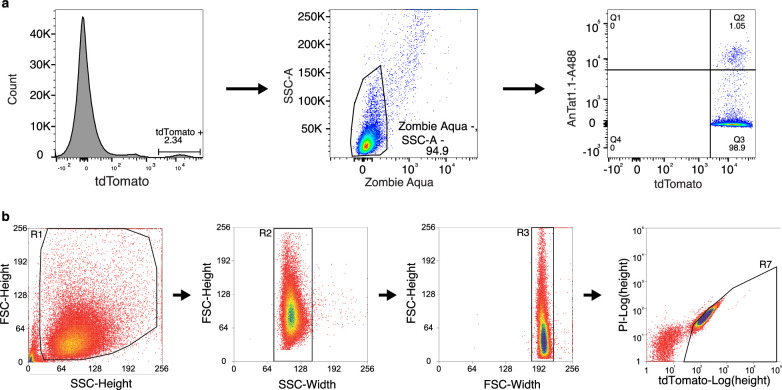
Flow cytometry and cell sorting gating strategies. (**a**) Example gating strategy for flow cytometry experiments from Fig. 3. Samples were first gated for tdTomato-positive cells, which represent *T. brucei* parasites expressing tdTomato in their cytoplasm. Then live cells were gated based on live/dead Zombie Aqua^TM^ staining. Finally, quadrants were placed around AnTat1.1-A488 positive and negative cells. (**b**) Gating strategy for single-cell sorting into 384-well plates for the SL-Smart-seq3xpress platform. This example is from the blood of mouse 2. Singlet parasites were selected and then tdTomato positive, PI (propidium iodide) negative *T. brucei* cells were sorted into single wells.

All samples, both blood and tissues, were stained with Zombie Aqua dye at 1:100 in PBS and washed with PBS following the manufacturer’s protocol (BioLegend, 423101). Samples were then stained for 10 min at 4 °C with a rabbit anti-AnTat1.1 polyclonal antibody diluted 1:15,000 in HMI-9 media and washed once with HMI-9 (antibody courtesy of J. Bangs^64^). Then, secondary antibody staining was performed while shaking for 10 min at 4 °C with Anti-Rabbit IgG (H+L), F(ab’)2 Fragment conjugated to Alexa Fluor 488 fluorescent dye (Cell Signaling Technology, 4412S). Finally, samples were washed with cold PBS and resuspended in PBS for flow cytometry analysis. Samples were run on a Beckton Dickenson A3 Symphony flow cytometer and analysis was performed using FlowJo (v.10.6.1) (see Extended Data Fig. 7a for the gating strategy).

### Immunofluorescence

Mice infected with AnTat1.1E chimeric triple reporter *T. brucei* parasites that express tdTomato were euthanized and perfused as previously described at days 6 and 13 postinfection. Lung, heart and gonadal fat were collected and fixed in 4% paraformaldehyde in PBS for 12 h at 4 °C. Postfixation, tissues were frozen, embedded in Optimal Cutting Temperature Compound (Tissue-Tek) and cut by cryostat microtome into 10 μm sections.

The following antibodies were applied to sections: rat antimouse CD31 (PECAM-1) (Santa Cruz Biotechnology no. sc-18916, 1:200) with goat antirat Fluor 488 (Cell Signaling Technology, no. 4416, 1:1,000). Coverslips were mounted using ProLong Gold (Life technologies). Tissues were imaged with ×4, ×10 and ×20 objectives using a Nikon Eclipse 90i fluorescence microscope (Nikon) and X-Cite 120 fluorescent lamp (Excelitas) with an ORCA-ER digital CCD camera (Hammamatsu) and ImageJ v.1.53 image analysis software. Image collection and analysis followed published guidelines for rigour and reproducibility^65^.

### Serum antibody ELISA quantification

Blood (25 μl) was collected by submandibular bleed on days 0, 6, 10 and 14 postinfection from mice infected with roughly five pleiomorphic EATRO1125 AnTat1.1E 90-13 *T. brucei* parasites. Serum was isolated using serum separator tubes (BD Microtainer SST tubes, 365967). IgM and IgG were quantified by enzyme-linked immunosorbent assay (ELISA) using ThermoFisher IgM and IgG kits following manufacturer protocols (IgG catalogue no. 88-50400-88, IgM catalogue no. 88-50470-88).

### Serum sample flow cytometry on *T. brucei*

Blood was collected from two mice infected with AnTat1.1E chimeric triple reporter *T. brucei* parasites, which initially express the VSG AnTat1.1, by cheek bleed. Serum was isolated by spinning blood samples at 10,000*g* for 5 min and pipetting off the top serum layer. EATRO1125 AnTat1.1E 90-13 *T. brucei* parasites^23^ expressing the VSG AnTat1.1 and Monomorphic Single Marker Lister427 VSG221 TetR T7RNAP bloodstream form (NR42011; Lot 61775530)^66^, which express VSG-2, were used for flow cytometry. Next, 10^6^ parasites were stained in duplicate while shaking for 10 min at 4 °C using mouse serum diluted 1:100 in PBS. As a positive control, a rabbit anti-AnTat1.1 polyclonal antibody diluted 1:15,000 in PBS was used following the same staining procedure (antibody courtesy of J. Bangs^64^). Then, secondary antibody staining was performed while shaking for 10 min at 4 °C with Antimouse IgG (H+L), F(ab’)2 Fragment conjugated to Alexa Fluor 647 fluorescent dye (Cell Signaling Technology, 4410S) or Anti-Rabbit IgG (H+L), F(ab’)2 Fragment conjugated to Alexa Fluor 647 fluorescent dye (Cell Signaling Technology, 4414S). Finally, samples were washed with cold PBS and resuspended in PBS for flow cytometry analysis. Samples were run on an Attune Nxt flow cytometer (Invitrogen) and analysis was performed using FlowJo (v.10.6.1).

### Single-cell sorting and RNA-seq library preparation

Blood and tissue samples (heart, gonadal fat and lung) from two mice were collected after perfusion as described above. For sorting parasites, the same tissue dissociation and flow cytometry was performed as described above, with the exception of viability staining, which was performed using propidium iodide instead of Zombie Aqua. Samples were kept on ice as much as possible through this process. Single live, tdTomato^+^ *T. brucei* cells were sorted into chilled 384-well plates for SL-Smart-seq3xpress library preparation containing lysis buffer and an RNA spike-in control using a Beckman Coulter MoFlo XDP cell sorter (see Extended Data Fig. 7b for the gating strategy). For each blood and tissue sample, a single plate of parasites were sorted for a total of 370 cells per sample.

The SL-Smart-seq3xpress library preparation approach was followed as described in ref. ^33^. In brief, single cells were lysed by incubation at 72 °C for 10 min in 0.3 µl of lysis buffer. Reverse transcription was done by adding 0.1 µl of reverse transcription mix to each well and incubation at 42 °C for 90 min, followed by ten cycles of 50 °C for 2 min and 42 °C for 2 min, with a final incubation at 85 °C for 5 min. Preamplification was done by adding 0.6 µl of a mix containing primers annealing to the Spliced-Leader sequence and to a conserved sequenced added by the oligodT primer during reverse transcription, with the following cycling conditions: 95 °C for 1 min, 16 cycles of: 98 °C for 10 s, 65 °C for 30 s, 68 °C for 4 min and finally 72 °C for 10 min. Following, the amplified cDNA was diluted by adding 9 µl of water per well. Next, 1 µl of each well was transferred to a new plate, 1 µl of tagmentation mix was added and the plate incubated at 55 °C for 10 min for tagmentation. The reaction was stopped by adding 0.5 µl of 0.2% SDS to each well and incubating for 5 min. The final index PCR was done by adding 1 µl of specific index primer combinations to each well and 1.5 µl of PCR mix. The following cycling conditions were used: 72 °C for 3 min, 95 °C for 30 s, 14 cycles of: 95 °C for 10 s, 55 °C for 30 s, 72 °C for 1 min; followed by 72 °C for 5 min. Single-cell libraries were then pooled and purified using AMPure XP beads at a ratio of 1:0.7. Libraries were run on a 4% non-denaturing PAGE gel and purified according to standard polyacrylamide gel purification protocols. Purified libraries from several plates were pooled and sequenced on a NextSeq 1000 sequencing platform to produce paired-end reads of 101 nucleotides (nt) (cDNA) and 19 nt (TAG + UMI read), and 8 nt for the index reads.

### Single-cell RNA-seq primary processing and VSG de novo assembly

The primary processing of the sequencing data was as described in ref. ^33^. In brief, the two reads containing the indexes (8 nt each) and the one containing the TAG + UMI (19 nt) were concatenated into a 35 nt read. Artefact reads containing the TAG sequence (or its reverse complement) in the cDNA reads were filtered out with Cutadapt^67^ (v.4.3).

For analysis of derepression by de novo assembly of VSGs, the filtered reads were sorted into individual files for each cell and these read files were run through our VSG-seq analysis pipeline individually using the same parameters as described above for bulk VSG-Seq. Using this approach, VSG open reading frames (ORFs) were assembled and quantified for each cell individually. VSG ORFs were then clustered among all single cells (VSG clusters were not related to previous VSG clusters from bulk VSG-seq and cannot be compared to the previous analysis based on cluster names).

For analysis of derepression by alignment to the genome, filtered reads were mapped with STAR^68^ (v.2.7.10a) to a hybrid fasta file combining the *T. brucei* EATRO1125 strain genome assembly (v.67, downloaded from TriTrypDB^69^) and the set of ten sequences used as RNA spike-in. The count matrix obtained was then corrected with the index hopping filtering pipeline scSwitchFilter, v.1.0.0 (https://github.com/colomemaria/scSwitchFilter). Only cells with at least 500 genes detected, 1,000 gene UMI transcript counts, 30 spike-in UMI counts and ten VSG UMI counts were used for downstream analyses. A total of 1,216 total cells fit these criteria out of 2,960 total cells sequenced. For each tissue and blood sample, a single plate (370 cells) was sequenced. For assessment of potential derepression (Extended Data Fig. 5b,e), VSGs with more than one UMI count were considered expressed in a cell. Cells were considered to be monogenically expressing a VSG (Extended Data Fig. 5e) if the VSG represented more than or equal to 80% of VSG UMI counts. Alignment and assembly data for each cell can be found in Supplementary Data 1–3.

For evaluating read coverage of VSGs and VSG clusters, Bowtie indexes were created for each reference sequence then reads from a single cell were aligned using Bowtie^53^ (v.1.2.3). Read coverage was calculated using deepTools^70^ (v.3.5.5) to convert BAM alignment files to bigWig coverage tracks. Coverage was then visualized using the ggcoverage^71^ package in R (https://github.com/showteeth/ggcoverage, v.1.2.0).

### Tsetse fly husbandry and fly bite-inoculated mouse infections

*Glossina morsitans morsitans* were maintained in the Yale School of Public Health insectary at 25 °C with 65–70% relative humidity under a 12 h:12 h light:dark photoperiod. All flies received defibrinated sheep blood (Lampire Biologicals) every 48 h through an artificial membrane feeding system^72^. Newly eclosed adult female flies were administered per os an initial blood meal containing 1 × 10^6^ per millilitre of *Trypanosoma brucei brucei* (strain RUMP 503; previously expanded in rats) and cysteine (10 µM; to increase the infection prevalence^73^). After this single parasite challenge, flies were maintained on normal blood every other day.

Thirty-five days postchallenge (the time it takes *T. b. brucei* to complete their developmental cycle within the tsetse fly and become infectious to a new vertebrate host), 6–8-week-old female C57Bl/6J mice were exposed to the bite of individual, trypanosome challenged flies 72 h after the flies had taken their last blood meal. Following the consumption of mouse blood, individual flies were microscopically dissected to confirm that their salivary glands were infected with vertebrate-infectious metacyclic stage *T. b. brucei* (if not, another fly was allowed to feed on the mouse until it was confirmed that an infectious fly had taken a blood meal). Five mice were infected using this method. All experiments using mice were performed in strict accordance with the Yale University Institutional Animal Care and Use Committee policies (Protocol 2014–07266 renewed on March 2023).

Once mice were infected, blood parasitaemia was counted by tail bleed every 2 days starting on day 4 postinfection by haemocytometer with a limit of detection of 2.22 × 10^5^ parasites per millilitre. Blood (25 μl) was collected by a submandibular bleed on days 6, 10 and 14 postinfection and placed into TRIzol LS. Five mice were anaesthetized and perfused at day 14 postinfection. Mice were perfused with 50 ml of PBS glucose (0.055 M d-glucose) with heparin. After perfusion, tissues were dissected and placed immediately into 1 ml of RNA Later. The heart, lungs, gonadal fat, subcutaneous fat, brain and skin (ear) were collected. Sequencing libraries were prepared and analysed following the methodology described above.

To quantify the mVSG repertoire of RUMP 503, we also collected a pool of tsetse saliva containing RUMP 503 *T. brucei* parasites. This sample was stored in TRIzol LS, RNA was extracted and the sample was prepared for sequencing as described above. The VSG-seq pipeline was used to quantify mVSG expression in the sample.

### Statistics and figures

Normality was tested for all Students *t*-tests and Dunnet’s tests analyses and can be found in the code on the repository at https://github.com/mugnierlab/Beaver2022. Nearly all VSG diversity measurements were found to be normally distributed, except for some samples with low VSG counts or sample numbers. We thus assumed normality for all VSG diversity measurements. All reported *P* values have been corrected for multiple comparisons using the Benjamini–Hochberg procedure. For all figures with boxplots, the box represents the first (25%) and third (75%) quartiles with a line at the median. Extending lines represent the maximum and minimum values not including outliers that are further than 1.5 times the interquartile range from either end of the box.

### Reporting summary

Further information on research design is available in the Nature Portfolio Reporting Summary linked to this article.

## Online content

Any methods, additional references, Nature Portfolio reporting summaries, source data, extended data, supplementary information, acknowledgements, peer review information; details of author contributions and competing interests; and statements of data and code availability are available at 10.1038/s41586-024-08151-z.

## Supplementary information

Reporting Summary

Supplementary Data 1 A summary of VSG expression in each cell by both genome alignment and de novo assembly, including categorization for Extended Data Fig. 5e.

Supplementary Data 2 The EATRO1125 VSG read alignment in each cell that meets quality control cut-offs (at least 500 genes detected, 1,000 gene UMI counts, 30 spike-in UMI counts and ten VSG UMI counts) for VSGs to which at least one VSG UMI is mapped. Raw alignment count tables and VSG count tables that include unfiltered cells can also be found at https://github.com/mugnierlab/Beaver2022.

Supplementary Data 3 The results from de novo assembly of VSGs in each cell. The Trinity name for each ORF and the top VSG blast hit for each assembled VSG ORF is reported, including the proportion of VSG expression accounted for by that VSG. For each cell, a dominant VSG is identified if it represented more than 80% of the VSG expressed in the cell and ‘NA’ denotes that there is not a dominant VSG in that cell. VSG assembly was attempted in all cells without any previous quality control filtering. Here, all VSG assemblies are reported without further filtering and the sequences can be found at https://github.com/mugnierlab/Beaver2022.

## Source data

Source Data Fig. 1

Source Data Fig. 2

Source Data Fig. 3

Source Data Fig. 4

Source Data Fig. 5

Source Data Extended Data Fig. 3

Source Data Extended Data Fig. 4

Source Data Extended Data Fig. 5

Source Data Extended Data Fig. 6

## Figures and Tables

**Extended Data Table 1:**
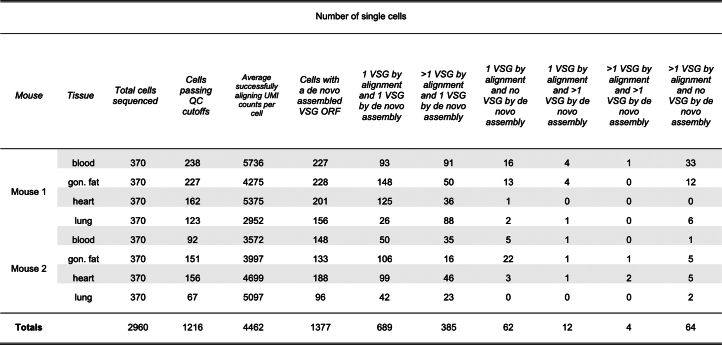
Summary of single cell sequencing results. *T. brucei* single cells were sorted from blood and tissues on day 14 post-infection from 2 mice and sequenced using the SL-Smart-seq3xpress platform

For de novo assembly of VSGs, all cells were considered without filters, but for all other columns only cells that had at least 500 genes detected, 1000 gene UMI counts, 30 spike-in UMI counts, and 10 VSG UMI counts were considered. Additionally, only VSGs that had >1 UMI counts when aligning to the EATRO1125 genome were considered to be detected.

**Extended Data Table 2:**
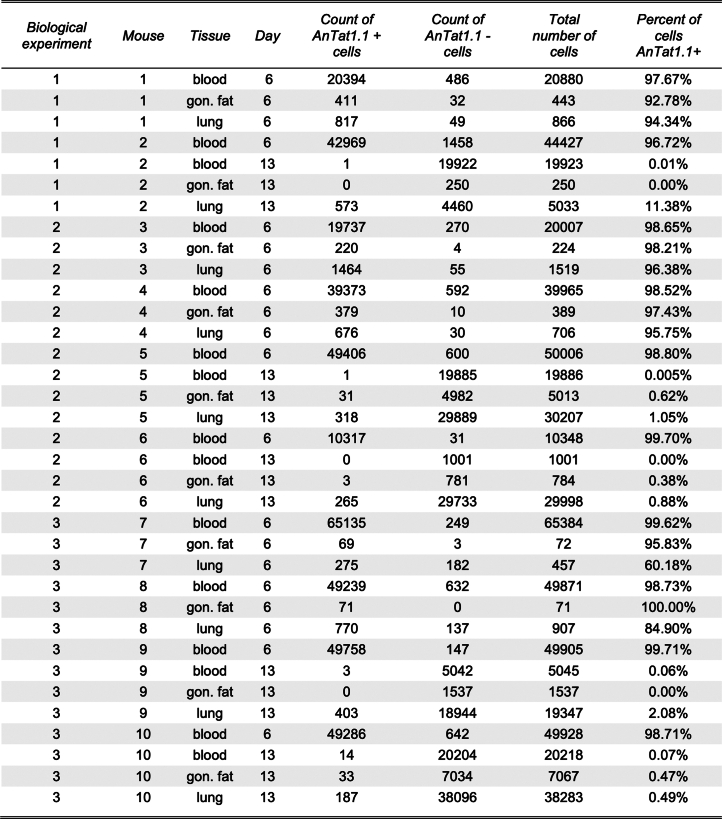
Summary table of the flow cytometry counts of tdTomato-positive parasites from infections with triple-marker *T. brucei* parasites stained with an anti-AnTat1.1 antibody

Parasites were collected from the blood and dissociated tissues of 10 mice on either day 6 or 13 post-infection and stained with an anti-AnTat1.1 polyclonal antibody. Here we report the raw number of live tdTomato-positive parasites that were Antat1.1 positive and negative in each sample. These data were used to generate Fig. 3b.

## Data Availability

Data for generating the analysis and figures in this paper are available at GitHub (https://github.com/mugnierlab/Beaver2022/) and Zenodo (10.5281/zenodo.13684001)^74^. The EATRO1125 genome used for single-cell RNA-seq analysis is available from TriTrypDB (version 67, available at https://w1.tritrypdb.org/common/downloads/release-67/TbruceiEATRO1125/fasta/data/TriTrypDB-67_TbruceiEATRO1125_Genome.fasta). Raw sequencing data are available in National Center for Biotechnology Information (NCBI) Sequence Read Archive under accession number PRJNA858046. Source data are provided with this paper.

## Code availability

All code used to perform the analysis and generate figures in this paper is available at GitHub https://github.com/mugnierlab/Beaver2022 and Zenodo (10.5281/zenodo.13684001)^75^.
