# Publisher Correction: Tissue spaces are reservoirs of antigenic diversity for *Trypanosoma brucei*

**DOI:** 10.1038/s41586-024-08389-7

**Published:** 2024-11-16

**Authors:** Alexander K. Beaver, Zhibek Keneskhanova, Raúl O. Cosentino, Brian L. Weiss, Erick O. Awuoche, Gretchen M. Smallenberger, Gracyn Y. Buenconsejo, Nathan P. Crilly, Jaclyn E. Smith, Jill M. C. Hakim, Bailin Zhang, Bryce Bobb, Filipa Rijo-Ferreira, Luisa M. Figueiredo, Serap Aksoy, T. Nicolai Siegel, Monica R. Mugnier

**Affiliations:** 1 Department of Pathology, Johns Hopkins School of Medicine, Baltimore, MD USA; 2 Department of Molecular Microbiology and Immunology, Johns Hopkins Bloomberg School of Public Health, Baltimore, MD USA; 3 Division of Experimental Parasitology, Faculty of Veterinary Medicine, Ludwig-Maximilians-Universität München, Munich, Germany; 4 Biomedical Center Munich, Division of Physiological Chemistry, Faculty of Medicine, Ludwig-Maximilians-Universität München, Munich, Germany; 5 Department of Epidemiology of Microbial Diseases, Yale School of Public Health, New Haven, CT USA; 6 Division of Infectious Diseases and Vaccinology, Berkeley Public Health Molecular and Cell Biology Department, Berkeley, CA USA; 7 Gulbenkian Institute for Molecular Medicine, Lisboa, Portugal

**Keywords:** Next-generation sequencing, Gene expression, Parasitic infection, Parasite immune evasion, Immune evasion

Correction to: *Nature* 10.1038/s41586-024-08151-z Published online 30 October 2024

This article was originally published under standard Springer Nature license (© The Author(s), under exclusive licence to Springer Nature Limited). It is now available as an open-access paper under a Creative Commons Attribution 4.0 International license, © The Author(s). In addition, the state abbreviation in affiliation 6 was shown as CT instead of CA. The errors have been corrected in the HTML and PDF versions of the article.

