# Supplementary material for: Tissue spaces are reservoirs of antigenic diversity for Trypanosoma brucei
Source: Nature. 2024 Oct 30;636(8042):430–7. doi: 10.1038/s41586-024-08151-z (PMC11634766; doi:10.1038/s41586-024-08151-z)
Supplement: Supplementary file 1 — Reporting Summary [file 41586_2024_8151_MOESM1_ESM.pdf]

nature portfolio

Corresponding author(s): Monica R Mugnier

Last updated by author(s): Jul 18, 2024

Reporting Summary

Nature Portfolio wishes to improve the reproducibility of the work that we publish. This form provides structure for consistency and transparency in reporting. For further information on Nature Portfolio policies, see our Editorial Policies and the Editorial Policy Checklist.

Statistics

For all statistical analyses, confirm that the following items are present in the figure legend, table legend, main text, or Methods section.

n/a	Confirmed	
☐	☒	The exact sample size (*n*) for each experimental group/condition, given as a discrete number and unit of measurement
☐	☒	A statement on whether measurements were taken from distinct samples or whether the same sample was measured repeatedly
☐	☒	The statistical test(s) used AND whether they are one- or two-sided<br>*Only common tests should be described solely by name; describe more complex techniques in the Methods section.*
☐	☒	A description of all covariates tested
☐	☒	A description of any assumptions or corrections, such as tests of normality and adjustment for multiple comparisons
☐	☒	A full description of the statistical parameters including central tendency (e.g. means) or other basic estimates (e.g. regression coefficient) AND variation (e.g. standard deviation) or associated estimates of uncertainty (e.g. confidence intervals)
☐	☒	For null hypothesis testing, the test statistic (e.g. *F*, *t*, *r*) with confidence intervals, effect sizes, degrees of freedom and *P* value noted<br>*Give P values as exact values whenever suitable.*
☐	☒	For Bayesian analysis, information on the choice of priors and Markov chain Monte Carlo settings
☐	☒	For hierarchical and complex designs, identification of the appropriate level for tests and full reporting of outcomes
☒	☐	Estimates of effect sizes (e.g. Cohen's *d*, Pearson's *r*), indicating how they were calculated

Our web collection on statistics for biologists contains articles on many of the points above.

Software and code

Policy information about availability of computer code

Data collection	For VSG-Seq, Sequencing libraries were sequenced with 100bp single-end reads on an Illumina HiSeq 2500. For single cell sequencing, samples were sequenced on a NextSeq 1000 sequencing platform to produce paired-end reads of 101nt (cDNA) and 19nt (TAG+UMI read), and 8nt for the index reads.
Data analysis	Sequencing data was analyzed using the VSG-Seq pipeline available on github (github: https://github.com/mugnierlab/VSGSeqPipeline). The following software and versions were used in the pipeline: Trinity (2.8.5), Biopython (1.72), Blast (2.9), Bedtools (2.29.2), cd-hit (4.8.1), trim-galore (0.6.4), bowtie(v1.2.3) and samtools (1.9). Data analysis was done in R (version 4.3.1). All code to generate figures and statistics can be found on github at https://github.com/mugnierlab/Beaver2022 (doi: 10.5281/zenodo.13684001). Analysis to investigate the existence of tissue-specific VSGs used HMMer (v3.1b2) Single cell data were analyzed using STAR (version 2.7.10a), Cutadapt (v4.3), and scSwitchFilter", v1.0.0 (https://github.com/colomemaria/scSwitchFilter. Expression matrices were analyzed in R (version 4.3.1). VSG coverage analysis was performed using Flow cytometry data was analyzed using bowtie (v1.2.3) deepTools (v3.5.5) and ggcoverage(v1.2.0) FlowJo (version 10.6.1).

For manuscripts utilizing custom algorithms or software that are central to the research but not yet described in published literature, software must be made available to editors and reviewers. We strongly encourage code deposition in a community repository (e.g. GitHub). See the Nature Portfolio guidelines for submitting code & software for further information.

Data

Policy information about availability of data

All manuscripts must include a data availability statement. This statement should provide the following information, where applicable:

- Accession codes, unique identifiers, or web links for publicly available datasets
- A description of any restrictions on data availability
- For clinical datasets or third party data, please ensure that the statement adheres to our policy

Data for generating the analysis and figures in this paper are available at https://github.com/mugnierlab/Beaver2022 (doi:10.5281/zenodo.13684001). The EATRO1125 genome used for scRNA-seq analysis is available from TriTrypDB (version 67, available at https://w1.tritrypdb.org/common/downloads/release-67/TbruceiEATRO1125/fasta/data/TriTrypDB-67_TbruceiEATRO1125_Genome.fasta). Raw sequencing data are available in National Center for Biotechnology Information (NCBI) Sequence Read Archive under accession number PRJNA858046.

Research involving human participants, their data, or biological material

Policy information about studies with human participants or human data. See also policy information about sex, gender (identity/presentation), and sexual orientation and race, ethnicity and racism.

Reporting on sex and gender	n/a
Reporting on race, ethnicity, or other socially relevant groupings	n/a
Population characteristics	n/a
Recruitment	n/a
Ethics oversight	n/a

Note that full information on the approval of the study protocol must also be provided in the manuscript.

Field-specific reporting

Please select the one below that is the best fit for your research. If you are not sure, read the appropriate sections before making your selection.

☒ Life sciences ☐ Behavioural & social sciences ☐ Ecological, evolutionary & environmental sciences

For a reference copy of the document with all sections, see nature.com/documents/nr-reporting-summary-flat.pdf

Life sciences study design

All studies must disclose on these points even when the disclosure is negative.

Sample size	Sample sizes were chosen based on preliminary data and for experiment feasibility. We chose four mice per time-point and collected tissues that have been shown to contain extravascular T. brucei. Two mice were infected and used for single-cell sequencing. For flow cytometry experiments, 5 mice were infected. For tsetse-initiated infections 5 mice were used as well. In single cell sequencing experiments, blood and tissue samples from 2 mice were collected and sequenced.
Data exclusions	For VSG-Seq, we excluded four samples (3 brain and 1 heart samples) because fewer than 100,000 reads aligned to VSG. For single-cell sequencing data, only cells which had 500 genes detected, 1000 gene UMI transcript counts, 30 spike-in UMI counts, and 10 VSG UMI counts were used for downstream analyses.
Replication	VSG-seq experiments in witdtype mice were performed using 4 independent biological replicates per time point over two independent experiments. VSG-seq experiments in AID-/- were performed using 2 (day 6) or 3 (day 14) biological replicates in one independent experiment. The overall result in wildtype mice was also replicated by repeating this experiment using a differnet parasite strain and a different infection route. This tsetse replication experiment was performed only once. The scRNA-seq experiment was performed twice, with 2 biological replicates per experiment. The two experiments showed similar results. Flow cytometry experiments were performed using 5 biologically independent replicate mice per timepoint in one experiment. The ELISA experiment and flow cytometric analysis of anti-VSG antibody were performed using 2 biologically independent animals examined over one independent experiment. The immunohistochemistry experiments were performed on 3 biologically independent animals over one experiment and representative images are shown.
Randomization	For all experiments, mice were assigned to each collection timepoint randomly, using a random number generator; mice were not housed by group with the exception for AID-/- and WT groups where each strain was in their own cages. Collected samples were prepared in batches based on tissue type. For VSG-seq experiments, all samples from an individual mouse were sequenced on the same flow cell. Single-cell sequencing plates were prepared in two batches and sequenced between two different runs.

Blinding	Blinding samples was not necessary for this study as the code used to analyze data was the same regardless of sample type. All measures were quantitative such that knowledge of sample groups could not influence the analysis.

Reporting for specific materials, systems and methods

We require information from authors about some types of materials, experimental systems and methods used in many studies. Here, indicate whether each material, system or method listed is relevant to your study. If you are not sure if a list item applies to your research, read the appropriate section before selecting a response.

Materials & experimental systems

n/a	Involved in the study	
☐	☒	Antibodies
☐	☒	Eukaryotic cell lines
☒	☐	Palaeontology and archaeology
☐	☒	Animals and other organisms
☒	☐	Clinical data
☒	☐	Dual use research of concern
☒	☐	Plants

Methods

n/a	Involved in the study	
☒	☐	ChIP-seq
☐	☒	Flow cytometry
☒	☐	MRI-based neuroimaging

Antibodies

Antibodies used	We used a custom rabbit anti-AnTat1.1 polyclonal antibody courtesy of Jay Bangs. In addition, the following commercially available antibodies were used: Zombie Aqua™(BioLegend, 423101), propidium iodide (BD, BDB556463), Anti-mouse IgG (H+L), F(ab')2 Fragment conjugated to Alexa Fluor® 647 fluorescent dye (Cell Signalling Technology, 4410S) or Anti-Rabbit IgG (H+L), F(ab')2 Fragment conjugated to Alexa Fluor® 647 fluorescent dye (Cell Signaling Technology, 4414S) , Alexa Fluor® 488 fluorescent dye (Cell Signaling Technology, 4412S), thermoFisher IgM and IgG kits following manufacturer protocols (IgG cat# 88-50400-88, IgM cat# 88-50470-88), and rat anti-mouse CD31 (PECAM-1) (Santa cruz biotechnology, cat# sc-18916) with goat anti-rat Fluor 488 (Cell signaling technology cat #4416).
Validation	We validated our anti-AnTat1.1 polyclonal antibody by flow cytometry on in vitro T. brucei cells expressing AnTat1.1 and T. brucei cells not expressing AnTat1.1 as a negative control. Live dead Zombie Aqua and propidium iodide staining was validated using live T. brucie cells and heat killed T. brucei cells.

Eukaryotic cell lines

Policy information about cell lines and Sex and Gender in Research

Cell line source(s)	*EATRO 1125 AnTat1.1E 90-13 T. brucei (gifted from Keith Mathews, Ref: Engstler, M. & Boshart, M. Cold shock and regulation of surface protein trafficking convey sensitization to inducers of stage differentiation in Trypanosoma brucei. Genes Dev. 18, 2798 (2004)).<br>*AnTat1.1E chimeric triple reporter T. brucei (from Brice Rotureau; Ref: Calvo-Alvarez, E., Cren-Travaillé, C., Crouzols, A. & Rotureau, B. A new chimeric triple reporter fusion protein as a tool for in vitro and in vivo multimodal imaging to monitor the development of African trypanosomes and Leishmania parasites. Infect. Genet. Evol. 63, 391–403 (2018)) .<br>*Monomorphic Single Marker Lister427 VSG221 TetR T7RNAP bloodstream form (NR42011; LOT: 61775530) Wirtz, E., Leal, S., Ochatt, C. & Cross, G. A. M. A tightly regulated inducible expression system for conditional gene knock-outs and dominant-negative genetics in Trypanosoma brucei. Mol. Biochem. Parasitol. 99, 89–101 (1999).<br>*Trypanosoma brucei brucei strain RUMP 503 supplied by Serap Aksoy.
Authentication	EATRO 1125 AnTat1.1E 90-13 and Lister427 T. brucei were authenticated by PCR of drug resistence markers and growth in drug selection. AnTat1.1E chimeric triple reporter T. brucei cells were validated by the growth in drug selection and by flow cytometry showing AnTat1.1 staining and tdTomato fluorescence.
Mycoplasma contamination	Cell lines were not tested for Mycoplasma contamination.
Commonly misidentified lines (See ICLAC register)	No commonly misidentified lines were used

Animals and other research organisms

Policy information about studies involving animals; ARRIVE guidelines recommended for reporting animal research, and Sex and Gender in Research

Laboratory animals	C57Bl/6J (WT, strain# 000664 Jackson Laboratory) or B6.129P2-Aicdatm1(cre)Mnz/J (AID-/-, strain# 007770 Jackson Laboratory) between 7-10 weeks old were used. Mice were infected by intravenous injection in the tail-vein or by tsetse-bite from flies with a confirmed salivary gland infection. 30-35 day old adult Glossina morsitans morsitans that had been fed a blood meal containing

infectious bloodstream form parasites at 2 days old were used to initiate infections.

Wild animals

No wild animals were used in this study.

Reporting on sex

Only female mice were infected and analyzed. This is standard in the field and some of our preliminary data suggested that sex does not have a major influence on antigenic variation dynamics in vivo. Financial constraints also limited our ability to evaluate sex-specific effects. Female flies were used for tsetse bite infections. This is because female flies live longer; it takes at least 30 days before transmissible salivary gland infections can be presumed, and most male flies do not survive this long.

Field-collected samples

No field-collected sample were used in this study.

Ethics oversight

All animal studies were approved by the Johns Hopkins Animal Care and Use Committee (protocol # MO22H163) and by Yale University Institutional Animal Care and Use Committee policies (Protocol 2014–07266 renewed on March 2023).

Note that full information on the approval of the study protocol must also be provided in the manuscript.

Plants

Seed stocks

Report on the source of all seed stocks or other plant material used. If applicable, state the seed stock centre and catalogue number. If plant specimens were collected from the field, describe the collection location, date and sampling procedures.

Novel plant genotypes

Describe the methods by which all novel plant genotypes were produced. This includes those generated by transgenic approaches, gene editing, chemical/radiation-based mutagenesis and hybridization. For transgenic lines, describe the transformation method, the number of independent lines analyzed and the generation upon which experiments were performed. For gene-edited lines, describe the editor used, the endogenous sequence targeted for editing, the targeting guide RNA sequence (if applicable) and how the editor was applied.

Authentication

Describe any authentication procedures for each seed stock used or novel genotype generated. Describe any experiments used to assess the effect of a mutation and, where applicable, how potential secondary effects (e.g. second site T-DNA insertions, mosiacism, off-target gene editing) were examined.

Flow Cytometry

Plots

Confirm that:

☒ The axis labels state the marker and fluorochrome used (e.g. CD4-FITC).

☒ The axis scales are clearly visible. Include numbers along axes only for bottom left plot of group (a 'group' is an analysis of identical markers).

☒ All plots are contour plots with outliers or pseudocolor plots.

☒ A numerical value for number of cells or percentage (with statistics) is provided.

Methodology

Sample preparation

Tissues were dissected and washed with HBSS (Hanks balanced salt solution, ThermoFisher Scientific 14175095). Tissue samples were minced and placed in DMEM (ThermoFisher Scientific, 11995065) containing either 1 mg/mL collagenase type 1 (ThermoFisher Scientific, 17100017) for adipose fat or 2 mg/mL collagenase type 2 (ThermoFisher Scientific, 17101015) for lung samples. Hearts were dissociated using 2 mg/mL collagenase type 2, 50U/mL DNase I, and 20U/mL Hyaluronidase. These were then incubated in a 37°C water bath for 1 hour and briefly vortexed every 10 minutes. Next, samples were passed through a 70µM filter and centrifuged at 2600 x g for 8 mins at 4 C, and the cell pellet was taken for antibody staining.
 Blood samples were collected by submandibular bleed and red blood cells were depleted by magnetic-activated cell sorting (MACS) with anti-Ter-119 MicroBeads (Miltenyi Biotech, 130-049-901) following the manufacturer's protocol. Cells were pelleted and washed with HMI-9 media. All samples, both blood and tissues, were stained with Zombie Aqua™ dye at 1:100 in PBS or with propidium iodide and washed with PBS following the manufacturer's protocol (BioLegend, 423101). Samples were then stained for 10 minutes at 4°C with a rabbit anti-AnTat1.1 polyclonal antibody diluted 1:15,000 in HMI-9 media and washed once with HMI-9 (antibody courtesy of Jay Bangs). Then, secondary antibody staining was performed while shaking for 10 minutes at 4°C. Finally, samples were washed with cold PBS and resuspended in PBS for flow cytometry analysis.

Instrument

Becton Dickenson A3 Symphony flow cytometer or for sorting, a Beckman Coulter MoFlo™ XDP cell sorter.

Software

FlowJo (version 10.6.1)

Cell population abundance

For single-cell sequencing, single cells were sorted into individual wells of a 384-well plate. Sample purity was not assessed because a T. brucei-specific library prep was performed, such that only T. brucei RNA would be sequenced in each library regardless of contamination.

Gating strategy

For flow cytometry experiments, we first gated on tdTomato positive cells, which represent T. brucei cells. We then gated on live cells based on live/dead controls. Finally, we gated AnTat1.1-A488 positive and negative cells. For single-cell sorting, Singlet parasites were selected and then tdTomato positive, PI (propidium iodide) negative T. brucei cells were sorted into single wells of a 384-well plate containing containing lysis buffer and an RNA spike-in control.

☒ Tick this box to confirm that a figure exemplifying the gating strategy is provided in the Supplementary Information.
